# Irradiation enhances the malignancy-promoting behaviors of cancer-associated fibroblasts

**DOI:** 10.3389/fonc.2022.965660

**Published:** 2022-10-19

**Authors:** Ziyue Zhang, Yi Dong, Bin Wu, Yingge Li, Zehui Liu, Zheming Liu, Yanjun Gao, Likun Gao, Qibin Song, Zhongliang Zheng, Yi Yao

**Affiliations:** ^1^ Cancer Center, Renmin Hospital of Wuhan University, Wuhan, China; ^2^ Hubei Provincial Research Center for Precision Medicine of Cancer, Wuhan, China; ^3^ Department of Oncology, Huang-gang Central Hospital, Huanggang, China; ^4^ College of Life Sciences, Wuhan University, Wuhan, China; ^5^ Department of Pathology, Renmin Hospital of Wuhan University, Wuhan, China

**Keywords:** cancer-associated fibroblasts, irradiation, lung cancer, activation, malignant, biological behavior

## Abstract

**Background:**

Cancer-associated fibroblasts (CAFs) are the important component of the tumor microenvironment (TME). Previous studies have found that some pro-malignant CAFs participate in the resistance to radiotherapy as well as the initiation and progression of tumor recurrence. However, the exact mechanism of how radiation affects CAFs remains unclear. This study aimed to explore the effect and possible mechanism of radiation-activated CAFs, and its influence on lung cancer.

**Methods:**

CAFs were isolated from surgical specimens *in situ* and irradiated with 8Gy x-rays. The changes in cell morphology and subcellular structure were observed. CAFs marker proteins such as FAP and α-SMA were detected by Western Blotting. Cell counting kit-8 (CCK8) assay, flow cytometry, wound healing assay, and transwell chamber assay was used to detect the activation of cell viability and migration ability. A nude mouse xenograft model was established to observe the tumorigenicity of irradiated CAFs *in vivo*. The genomic changes of CAFs after radiation activation were analyzed by transcriptome sequencing technology, and the possible mechanisms were analyzed.

**Results:**

The CAFs showed a disorderly growth pattern after X-ray irradiation. Subcellular observations suggested that metabolism-related organelles exhibited more activity. The expression level of CAFs-related signature molecules was also increased. The CAFs irradiated by 8Gy had good proliferative activity. In the (indirect) co-culture system, CAFs showed radiation protection and migration induction to lung cancer cell lines, and this influence was more obvious in radiation-activated CAFs. The radiation protection was decreased after exosome inhibitors were applied. Vivo study also showed that radiation-activated CAFs have stronger tumorigenesis. Transcriptome analysis showed that genes were enriched in several pro-cancer signaling pathways in radiation-activated CAFs.

**Conclusions:**

Our study confirmed that CAFs could be activated by ionizing radiation. Irradiation-activated CAFs could promote cancer cell proliferation, migration, radiotherapy tolerance, and tumorigenesis. These results suggested that irradiation-activated CAFs might participate in the recurrence of lung cancer after radiotherapy, and the inhibition of CAFs activation may be an important way to improve clinical radiotherapy efficacy.

## Introduction

In the past decades, advances in radiotherapy have improved overall survival for malignant tumors. However, primary resistance and acquired tolerance to radiotherapy remain significant challenges in clinical practice (1). Previous studies have found that bidirectional communication between cells and their microenvironment is critical for the malignant biological behavior of tumors (2–4).

As an important cellular component in the tumor stroma, cancer-associated fibroblasts (CAFs) can promote the malignant biological behavior of cancer cells. α-smooth muscle actin (α-SMA) (5), fibroblast specific protein-1 (FSP-1) (6), fibroblast activation protein (FAP) (7), and platelet-derived growth factor receptor-β (PDGFR-β) (8) are considered as traditional CAFs biomarkers. CAFs influence the chemotaxis of endothelial progenitor cells and monocytes (9) and promote survival (10), invasion and metastasis (11), and tumor angiogenesis (12) of cancer cells. Also, it has been suggested that CAFs can induce acquired drug resistance (13) and radiation resistance (14). In short, CAFs confer a mesenchymal-like phenotype and enhance metastasis of both premalignant and malignant epithelial cells, whereas normal fibroblasts promote an epithelial-like phenotype and suppress metastasis (15). CAFs have a diverse origin, which also leads to their heterogeneity; yet, they are mainly differentiated from normal fibroblast (NF) located close to cancer cells. When specific stimulation occurs, some NF can be activated into CAFs with different forms and functions, triggering various “pro-malignant” effects (16).

Previous studies on improving the efficacy of radiotherapy have mainly focused on cancer cells alone while ignoring the complex biological interactions between them and the tumor microenvironment (TME) (17). Numerous studies have suggested that radiation breaks the DNA strands of tumor cells and increases the secretion of transforming growth factor-β (TGF-β) and hypoxia-inducible factor 1-α (HIF-1α), thus suppressing the immune system and inducing radio-resistance (18).

Over the years, the variation and role of tumor stroma have attracted increasing attention during radiotherapy (19). Previous studies have found that some pro-malignant CAFs participate in the resistance to radiotherapy as well as the initiation and progression of tumor recurrence. In addition, a multivariate analysis showed that α-SMA/epithelial area ratio was an independent prognostic value associated with poor recurrence-free survival, suggesting that neoadjuvant treatment impacts on CAFs (20). Moreover, some CAFs activation following radiation led to altered growth factor secretion and release of numerous modulators of the ECM and cytokines, including TGF-β, which is a complex and pleiotropic cytokine that directly affects tumor cells and CAFs, driving HIF-1 signaling, reducing the activation of T-cells and dendritic cells (DCs), remolding the tumor microenvironment, and resulting in the progression of cancer (17).

The aim of this study was to explore the effect of radiation on the activation of CAFs *in vitro* and *in vivo* experiments and to further explore the influence of radiation-activated CAFs on the development of lung cancer and its possible mechanism.

## Materials and methods

### Materials and reagents

The antibodies used in this study were anti-GAPDH (60004-1-Ig, Proteintech, China), anti-α-SMA (14395-1-AP, Proteintech, China), anti-FAP (A11572, Abclonal, China), anti-VIM (60330-1-Ig, Proteintech, China), and anti-KI67 (27309-1-AP, Proteintech, China). Flow cytometry Kit (Multi Sciences, China), CCK8 reagent (Dojindo, Japan), MTT Reagent (Sigma, Germany), BCA1-1KT kit (Sigma, Germany), RIPA lysate (Beyotime, China), and inhibitor of exosome GW4869 (MCE, Shanghai) were purchased from Baitengruida Biotechnology. Optical microscope (Nikon, Japan), HT7800 Transmission Electron microscope (Hitachi, Japan), microplate reader (Bio-RAD, USA), and flow cytometry (BIO-RAD, USA) were provided by the central lab of Renmin Hospital of Wuhan University. The Elekta Infinity™ linear accelerator (Elekta, Sweden) was provided by Renmin Hospital of Wuhan University. Four-week-old female BALB/c nude mice were purchased from Weitonglihua Experimental Animal Technology.

### Primary CAFs isolation and cell culture

Primary CAFs were isolated and extracted from the lung cancer tissue of an adult male NSCLC patient who underwent surgical resection (the study was approved by the Ethics Committee of the Renmin Hospital of Wuhan University). The peritumoral tissue was taken from an area 1.0 cm away from the edge of lung cancer tissue, cleaned with 1% penicillin/streptomycin in PBS at 4°C, and cut into 1.0 mm^3^. The tissue was then put into the dish with 1 ml of fetal bovine serum, cultured in a CO_2_ incubator for 1 h, and then mixed by inverting the dish overnight. An appropriate amount of DMEM containing 10% fetal bovine serum was added to each dish after 24 h for upright culture, and the medium was changed every 3 days. When spindle cells migrated away from the edges of the tissue clumps and were fused to about 85%, the subculture was carried out. CAFs were used for further experiments after 5-8 passages.

CAFs were grown in Dulbecco’s Modified Eagle Medium (DMEM, HyClone, USA); MRC-5 cells were cultured in Eagle’s Minimal Essential Medium (EMEM, HyClone, USA); lung cancer cells A549 and H1299 were cultured in Roswell Park Memorial Institute 1640 Medium (RPMI-1640, HyClone, USA). All mediums were supplemented with 10% fetal bovine serum (FBS) and 1% penicillin/streptomycin. If not specified, all cells were maintained in a humidified atmosphere containing 5%CO_2_/95% air at 37°C. A549, H1299, and MRC5 cells were kind gifts from Wuhan University School of Life Sciences laboratory.

### Cell radiation and conditioned medium

Confluent CAF cultures were irradiated using a clinical 6MV X-ray beam produced by the Elekta Infinity™ linac. The source-to-surface distance was 100 cm with a dose rate of 300 MU/min. Except for where otherwise indicated, CAF received 8Gy, while A549 was 6Gy as reported previously (19). Conditioned medium (CM) was obtained as described below.

After conventional culture, the human lung adenocarcinoma cell line (A549) was divided into the irradiated group and the unirradiated group. Then these two groups were divided into three subgroups, respectively, by adding the supernatant of ordinary DMEM (blank), non-irradiated CAFs supernatant (CAF_0_-CM), and CAFs supernatant that underwent 8Gy X-ray irradiation (CAF8-CM) at a ratio of 7:5 (volume ratio). After 2 h of culture, the irradiated group was given a single dose of 6Gy X-ray at room temperature, and the pseudo-irradiated group was placed in the irradiation chamber for 1 min. The cells from both groups were cultured. The human NSCLC cell line H1299 and human embryonic lung fibroblast cell line MRC-5 were routinely cultured for the following experiments.

**Figure 1:**
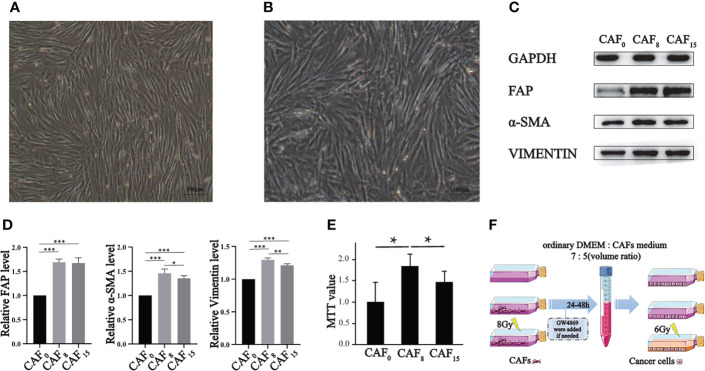
Effect of irradiation on the morphology, molecular markers, and proliferation of CAFs. **(A, B)** Morphology of CAFs without irradiation **(A)** and CAFs that received 8Gy irradiation **(B)** were compared under a light microscope (100×), and both groups of cells were spindle-shaped, but the irradiated cells were slightly disorganized; **(C)** Representative western blotting for FAP, α-SMA, and vimentin in CAFs that received 0Gy, 8Gy, and 15Gy irradiation; **(D)** The relative expression differences of the biomarkers after normalization of the GAPDH were analyzed by quantitatively comparing the density differences of the immunoblot bands; **(E)** MTT experiment was used to analyze the proliferation activity of CAFs under 0Gy, 8Gy, and 15Gy irradiation. T-test, *P < 0.05; **(F)** The sketch map of supernatants preparations of CAFs. (T-test, data are presented as the mean ± S.D. *P < 0.05, **P < 0.01, ***P < 0.001).

The primary cultured CAFs with or without irradiation were pretreated with GW4869 (10 μmol/L) for 48 h, and their supernatants were prepared at 4°C for later use. Each group was cultured in normal DMEM (blank), supernatant of GW4869 treated CAF_0_ (G+CAF_0_-CM), and supernatant of GW4869 treated CAF_8_ (G+CAF_8_-CM) with a ratio of 7:5 (volume ratio), respectively. After 2 h in the incubator, the irradiation group A549 was given X-ray irradiation at room temperature with a single dose of 6Gy, and the cells of the two groups continued to be cultured. The processes are detailed in **Figure 1F**.

### Morphology and subcellular structure observation of CAFs

After reaching a confluency of 60%, cells were treated with 8 Gy irradiation and then cultured for 8 h. The morphology and subcellular structure of CAFs were observed by an optical microscope. As for the transmission electron microscope, the collected cell sediment was fixed using 4% glutaraldehyde after centrifugation. This was further processed into ultra-thin slices of 60 nm in thickness, followed by observation for target subcellular structures like mitochondria, endoplasmic reticulum, etc.

### Western blotting

Protein lysates were obtained according to the previously described procedures (21). BCA1-1KT kit was used for the protein quantification assay. An appropriate amount of protein was mixed with 5× loading buffer and loaded on the 10% SDS-PAGE. After membranes were blocked with 5% non-fat milk, they were incubated with primary antibodies at 4°C overnight and appropriate horseradish peroxidase (HRP)-conjugated secondary antibodies at room temperature for 1.5 h. Protein bands were visualized using the enhanced chemiluminescence system. All experiments were performed three times. Protein levels were normalized as a ratio to GAPDH. The primary antibodies were: FAP (1:1000, Abclonal), Vimentin (1:50000, proteintech), and α-SMA (1:5000, proteintech).

### Cell apoptosis assay

Flow cytometry was used to analyze the apoptosis of A549 cells. The cells were seeded into a 6-well plate (1×10^6^/well) overnight. The medium was changed with a conditional medium 2 h before irradiation. After 24-hour incubation, cells in each group with different treatments were washed with PBS 3 times; the supernatant was discarded, digested with trypsin without EDTA, and then gently mixed with 500 μL binding buffer, 5μL Annexin V-FITC, and 5 μL PI. The cells were incubated at 4°C for 20 min under dark conditions and detected by flow cytometry.

### Cell viability assay

The viability of A549 cells was analyzed using CCK-8 kits. The cells were digested by trypsin and then suspended. Approximately 3.5×10^4^ cells per well were placed in 96-well plates and cultured for 24, 48, and 72 h, respectively. After each time point, a 10 μL of CCK-8 was added to each well at 37°C for 2 h. The absorbance at 450nm was determined using a microplate reader. Three wells were taken from each group to calculate the average value and draw the proliferation curve.

The viability of CAFs was analyzed using a MTT assay. The cells were seeded in 96-well plates (5×10^3^ cells/well) after irradiation and cultured for 24 h. Then, 20 μl MTT reagent was added to each well for 4 h. After removal of the medium, 150 μl of DMSO was added to each well and properly mixed for another 10 min. The absorbance at 570 nm was determined using a microplate reader. A blank well (medium, MTT, DMSO) was included for comparison. All experiments were performed three times.

### Wound healing assay

A549 cells were placed into 6-well plates (2.5×10^6^ cells/well). The two groups (irradiated and irradiated groups) were divided into three subgroups, respectively (see above). The irradiated group was given a single dose of 6Gy X-ray. After the cell reached 90% confluence, a line was drawn using a marker on the bottom of the dish, and then a sterile 1,000-μl pipet tip was used to scratch five separate wounds through the cells, moving perpendicular to the line. The cells were gently rinsed twice with PBS to remove floating cells and incubated in a fresh culture medium containing 1% FBS. Images of the scratches were taken by using an inverted microscope at ×10 magnification at 0, 12, 24, 36, and 48 h of incubation. Image J software (Version 1.53n) was used to calculate the percentage of migration area and draw the time-healing area curve. The percentage of migration area was quantitatively analyzed using cells migration area when measuring divided by the wound area at 0 h.

### Transwell migration assay

Transwell migration assay was performed using 24-well culture plates. Briefly, 500 μl of CAFs/MRC5 suspension (2×10^4^) was added to each well (lower chamber) overnight. The experimental group was given single X-ray irradiation of 8Gy, after which they were cultured for an additional 2 h. A549 or H1299 cells (5×10^4^/200μL) were seeded in the upper chamber and allowed to migrate the lower chamber. The cells were removed after co-culture for 24 h. A549 or H1299 cells passing through the polycarbonate membrane were observed and counted under the microscope after cleaning, fixed with 4% paraformaldehyde, and stained with crystal violet dye.

### Construction of xenografted mouse model

All animal studies (including the mice euthanasia procedure) were done in compliance with the regulations and guidelines of Renmin Hospital of Wuhan University institutional animal care and conducted according to the AAALAC and the IACUC guidelines. All the animals were housed in an environment with a temperature of 22 ± 1 °C, relative humidity of 50 ± 1%, and a light/dark cycle of 12/12 h.

Eighteen 4-week-old female BALB/c nude mice were divided into three groups. A549 cells (2×10^6^ cells), A549 and CAFs mixed cells (A549: CAF_0_, 1:1, a total of 4×10^6^ cells), and A549 and 8Gy irradiated CAFs mixed cells (A549: CAF_8_, 1:1, a total of 4×10^6^ cells) were subcutaneously inoculated under the right axilla of animals. The living status, body weight, and tumorigenesis of mice were observed and recorded daily. At the end of the animal experiments (about 25 days), the subcutaneous tumor tissue was completely removed. The long diameter (D) and short diameter (d) of the tumor size were measured. The tumor volume was calculated by V= d^2^ ×D/2, and the tumor volumetric time growth curve was plotted.

### Immunohistochemistry

The dissected subcutaneous tumor tissues were fixed using 4% paraformaldehyde. The expression of nuclear proliferation antigen ki67 and α-SMA in tumor tissues of each group was analyzed by immunohistochemistry. The experimental steps followed the previous standard protocols (22). In short, the tumor tissues of each group were fixed, embedded, sectioned, stained, and mounted, and then observed under a common light microscope. The collected pictures were calculated and analyzed by Image J software (Version 1.53n). The mean density was calculated and analyzed using the integrated density of the pre-processed images divided by the total area.

### RNA extraction and transcriptome sequencing

Primary cultured CAFs and 8Gy irradiated CAFs (CAF_8_) were used for RNA sequencing. Cells were treated as described above (see the materials and methods section). Then, cells were incubated for 24 h after irradiation and then collected. Total RNA was isolated using Trizol (Invitrogen, USA) according to the manufacturer’s instructions. By analyzing the RNA-seq raw data obtained from the HiSeq platform, we compared the differential expressed genes, and the enrichment analysis, including GO, and KEGG was further conducted. The differentially expressed genes that underwent GO and KEGG enrichment analyses with a q value < 0.05 were screened. The enrichment results were visualized by R software. Heat maps of differential oncogenic genes were visualized using “heatmap” R package.

### Statistical analysis

Data was statistically analyzed and plotted by GraphPad Prism (Version 8.0.1) and SPSS (Version 20.0). A two-tailed unpaired Student’s t-test was used for inter-group comparison. All data was represented as mean ± SD. Differences were considered statistically significant if *P*-value <  0.05.

## Results

### Activation of CAFs proliferation by ionizing radiation and its potential mechanism

Under a light microscope, CAFs showed a long spindle shape with dense growth and a slightly disordered and non-directional arrangement (**Figure 1A**). The contact and density inhibition were lost when reaching a certain density. Under 8Gy X-ray irradiation, the shape, size, and growth mode of CAFs did not significantly change (**Figure 1B**), thus suggesting that irradiation does not cause morphological changes to the cells.

Next, we detected intrinsic changes in cells at the protein level. Western blot showed that CAFs biomarkers were up-regulated with relatively low-dose irradiation (**Figure 1C**). FAP, vimentin and α-SMA were significantly increased after single irradiation of 8Gy and 15Gy compared with 0Gy. The expression level of α-SMA and vimentin under the 8Gy condition were slightly higher than under the 0Gy condition but not under the 15Gy condition (**Figure 1D**). The increased expression of the above cytoskeleton-associated proteins indicates that irradiated CAFs have an enhanced motor migration capacity, promoting an aggressive phenotype in metastasis.

To further explore the alteration of CAFs in other functional phenotypes after irradiation, we verified the proliferation alteration of CAFs by MTT assay. According to the MTT results, irradiation boosted the proliferative capacity of CAFs. The proliferation rate of CAFs irradiated with 8Gy and 15Gy was double that of 0Gy, and 1.5 times that of 0Gy, respectively (*P* < 0.001). CAFs exposed to 8Gy exhibited the highest proliferation capacity (**Figure 1E**). The above changes suggest that CAFs can exhibit malignant behavior after treated with 8Gy, which also imply that the CAFs organelles related to heredity, metabolism, and synthesis from the observation have been modified. The electron microscopic results further confirmed these changes.

**Figure 2:**
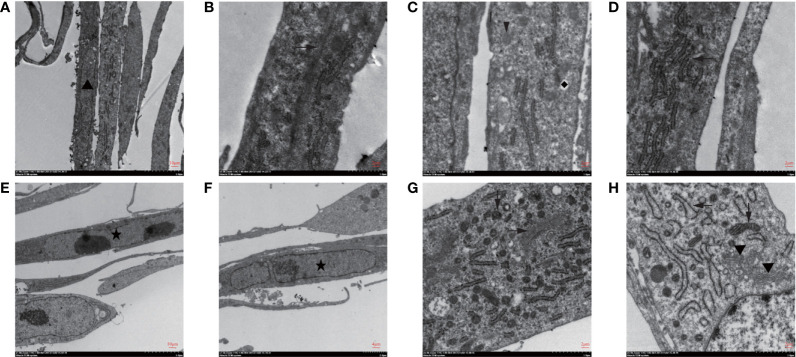
Subcellular structure of CAFs changed after irradiation was seen under transmission electron microscopy. **(A–D)** The observation of the subcellular structure of CAFs before irradiation showed that the nuclei were oval with obvious nucleoli (▲). Rough endoplasmic reticulum (**←**), mitochondria (↓), vesicles (◆), microtubules (→), and microfilaments can be seen in the cytoplasm; **(E–H)** The observation of the subcellular structure of CAFs after 8Gy irradiation revealed double nucleoli and double nuclei (★) and lobulated nuclei in the nucleus. The rough endoplasmic reticulum increased and expanded, and mitochondria were divided and increased, while Golgi apparatus (▼) increased in the cytoplasm.

There was no significant change in subcellular morphology after ionizing radiation; yet, double nucleoli, double nuclei, and lobulated nuclei were found in CAFs (**Figures 2A, E, F**). The rough endoplasmic reticulum increased and expanded in the cytoplasm (**Figures 2D, H**). As shown in **Figures 2B, G**, the microtubules increased in bundles. Mitochondria fission and Golgi apparatus also showed an increased status (**Figures 2C, G, H**).

Several investigations have reported that low-dose ionizing radiation could enhance cell proliferation *via* transient ERK1/2 and p38 activation in normal human lung fibroblasts (23). The phenotype of irradiated CAFs did reveal its activation, and the transcriptome data also provided an insight into the potential mechanism of activation of CAFs when exposed to 8Gy. Ionizing radiation activated multiple proliferation-related and cancer-promoting signaling pathways in CAFs.

**Figure 3:**
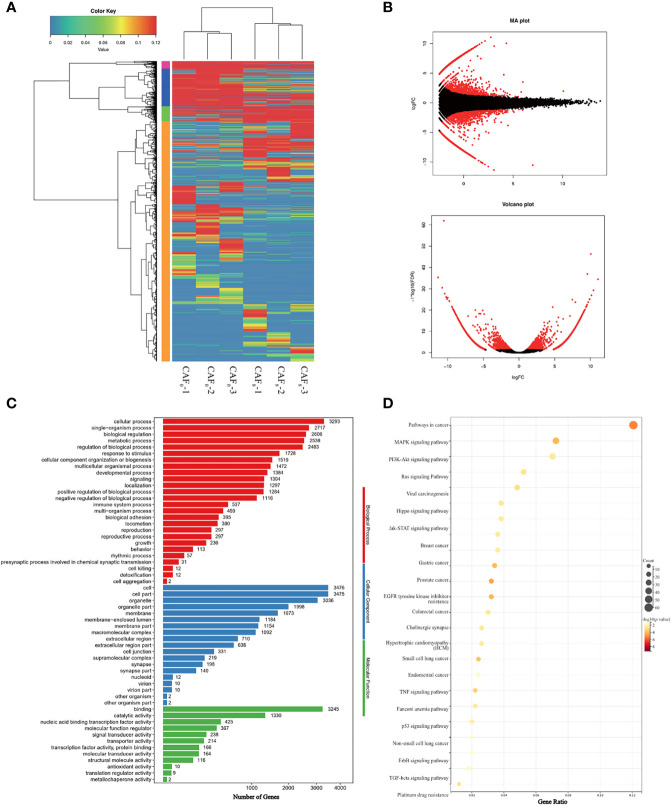
Identification and functional characterization of CAFs under 0Gy and 8Gy irradiation. **(A)** The cluster analysis of 0Gy compared to 8Gy irradiated CAFs (3 samples for each group) and the heat map showed that the irradiated CAFs differed more significantly at the gene level; **(B)** Volcano plot of differential genes after 0Gy vs. 8Gy irradiation of CAFs; **(C)** Gene Ontology (GO) analysis of differentially expressed genes; **(D)** Kyoto Encyclopedia of Genes and Genomes (KEGG) analysis of differentially expressed genes.

Differentially expressed analysis showed that large amounts of oncogenic genes were up-regulated (fold-change>2, FDR<0.05) (**Figure 3A**). Genes associated with cell proliferation like MITF, ABL2, and XIAP, and oncogenes like PDGFB and ERG were highly expressed in CAF_8_. The enrichment of Gene Ontology (GO) further showed that differentially expressed oncogenes were mainly enriched in cell proliferation, regulation of cell proliferation, positive regulation of the biological process, and positive regulation of cellular process (**Figure 3B**). The Kyoto Encyclopedia of Genes and Genomes (KEGG) analysis showed that differentially expressed oncogenes were enriched in pathways in cancer, Ras signaling pathway, PI3K-Akt signaling pathway, MAPK signaling pathway, and TGF-β and other signaling pathways (**Figure 3C**). To sum up, this data confirms that an irradiation dose of 8Gy can promote malignant transformation.

### CAFs promote the deterioration of lung cancer cells after ionizing irradiation

**Figure 4:**
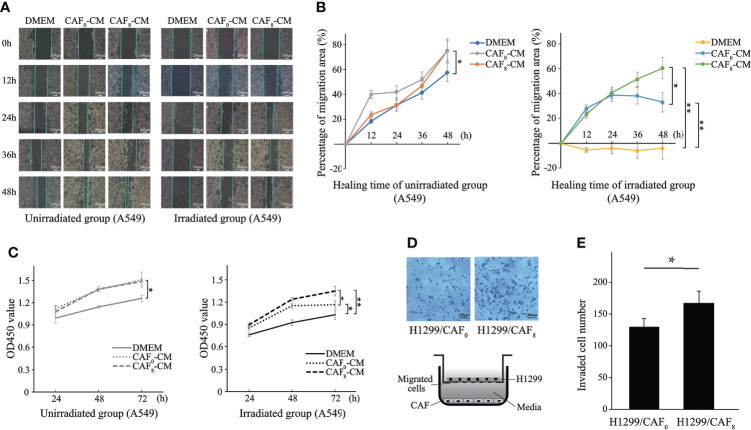
CAFs’ conditioned medium (CM) promoted proliferation and migration of lung cancer cells after ionizing irradiation. **(A)** Representative images of wound healing assay for the A549 cells in irradiated group and unirradiated group treated with DMEM, CAF_0_-CM, and CAF_8_-CM; **(B)** Quantification of the change in healing area (%) over time in different subgroups; **(C)** Differences in the proliferation of A549 after irradiation with or without 6Gy in different conditioned medium were analyzed by the CCK8 assay; **(D)** Transwell assay analyzed the migration of H1299 cells co-cultured with CAF_0_ and CAF_8_; **(E)** The histogram showed the number of transmembrane H1299 cells in two groups. (T-test, data are presented as the mean ± S.D. *P < 0.05, **P < 0.01).

Clinical practice has revealed the presence of tumor recurrence in patients treated with low radiation doses and that the TME has an important role in this process (14). This study further explored whether activated CAFs are involved in this process. The wound healing assay demonstrated that CAFs enhance the proliferation of A549 cells after ionizing irradiation. The wound healed faster in the non-irradiated A549 cells of the CAF_0_-CM and CAF_8_-CM subgroups compared to the DMEM subgroup (*P* < 0.05) (**Figures 4A, B**). After 72 h, the wounds of the CAF_0_-CM and CAF_8_-CM subgroups were nearly completely healed. In the irradiation group, the wound healing of all subgroups slowed down; it was almost stationary in the DMEM subgroup, while it was faster in the CAF_8_-CM subgroup than that of the CAF_0_-CM subgroup (*P* < 0.05). Thus, we concluded that CAFs could promote the migration of A549 or radiation-damaged A549, and this ability could be enhanced by irradiated-activated CAFs.

To further investigate the effect of CAFs on lung cancer cells (A549), we conducted the CCK8 assay to detect their effect on the proliferation of A549. As shown in **Figure 4C**, the proliferation capacity of A549 cells irradiated by 6Gy X-ray (solid black line) was significantly lower than 0Gy (solid gray line). However, the number of A549 cells in the CAF_0_-CM subgroup (gray dotted line) and CAF_8_-CM subgroup (gray dash line) was significantly higher than in the DMEM group (solid gray line) after 72 h in the unirradiation group (0Gy). From the proliferation rate, the slope within 72 h of the CAF_0_-CM subgroup (gray dotted line) and CAF_8_-CM subgroup (gray dash line) was similar but significantly higher than the control (*P* < 0.05). Under 6Gy irradiation, the CAF_0_-CM subgroup (black dotted line) and the CAF_8_-CM subgroup (black dash line) showed a similar proliferation trend to the A549 cells of the DMEM irradiation group. The proliferation rate of the CAF_8_-CM subgroup was the fastest within 48 h, while the slope of the CAF_0_-CM subgroup (black dotted line) decreased rapidly after 48 h and showed the inhibition of proliferation. All these analyses revealed that irradiated CAFs have a stronger promotion effect on A549 cell proliferation.

Apart from the adenocarcinoma, another non-small cell lung cancer cell line, H1299, was also applied for validation. CAFs had a stronger inductive effect on the migration ability of lung cancer cells H1299 by transwell cell co-culture experiment. H1299 cells immersed in irradiated CAFs (CAF_8_) culture medium had a higher number and density of transmembrane cell clones (*P* < 0.05) compared with un-irradiated CAFs (CAF_0_) (**Figures 4D, E**). The number of transmembrane cell clones in the H1299/CAF_8_ subgroup was about 20% more than that in H1299/CAF_0_ subgroup.

**Figure 5:**
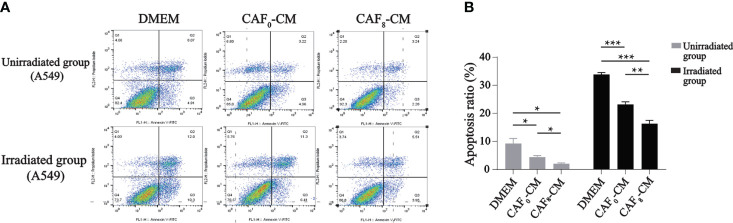
Flow cytometry analyzed the apoptosis of A549 cells received irradiation or not and treated with DMEM, CAF_0_-CM, CAF_8_-CM. **(A)** CAFs exhibited an inhibitory effect on A549 cells apoptosis, which showed more obvious in radiation-activated CAFs. In the unirradiated group, the CAF_8_-CM subgroup displayed more normal cells with less viable apoptotic cells. And when A549 received 6Gy irradiation, the number of the viable apoptotic cells in DMEM subgroup was twice as many as that in the CAF_8_-CM subgroup, which indicated the protective value of CAFs for irradiated A549; **(B)** A quantitative statistical analysis of the apoptotic cells in the different groups was carried out, and the bar graphs visualized the differences in apoptosis. (T-test, data are presented as the mean ± S.D. *P < 0.05, **P < 0.01, ***P < 0.001).

Flow cytometry results showed that irradiated CAFs had a stronger inhibitory effect on apoptosis of A549 cells (**Figure 5**). In the unirradiated A549 cell group, the apoptosis rate of the CAF_0_-CM and CAF_8_-CM subgroups was about 50% and 80% lower than the DMEM control (*P* < 0.05). In the irradiation group, the apoptosis rate of the CAF_0_-CM subgroup and CAF_8_-CM subgroup showed the same inhibitory trend (*P* < 0.05).

**Figure 6:**
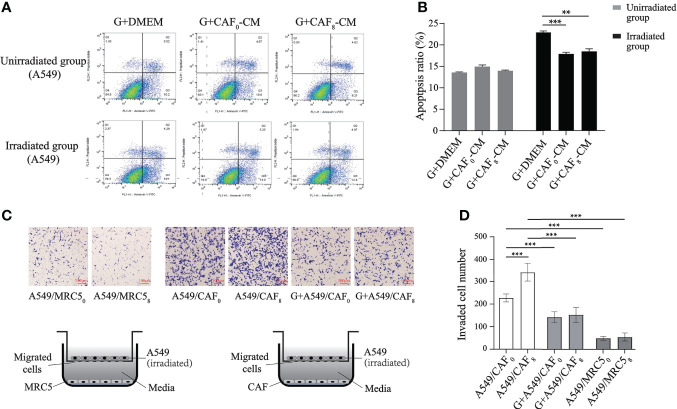
Exosome inhibitor GW4869 (G) inhibited the proliferation and migration of A549 cells induced by irradiated CAFs. **(A)** Flow cytometry analyzed the apoptosis of A549 cells received irradiation or not and treated with G+DMEM, G+CAF_0_-CM, and G+CAF_8_-CM. GW4869 (exosome releasing inhibitor) can inhibit “apoptosis protection” effect of CAFs on A549 cells in unirradiated group almost completely, but partially in irradiated group; **(B)** The histogram of apoptosis ratio (%) of the A549 cells in different subgroups of A; **(C)** Designation of transwell assay and the results of transmembrane A549 cells when co-cultured with MRC5_0_, MRC5_8_, CAF_0_ ± GW4869, CAF_8_ ± GW4869. Radiation-activated CAFs promoted migration of lung cancer cell, which can be inhibited by GW4869. All A549 cells received 6Gy irradiation before seeding on the upper chamber and co-cultured with CAFs or MRC5; **(D)** Statistics histogram of the numbers of invaded cells of different subgroups in **(C)**. (T-test, data are presented as the mean ± S.D. **P < 0.01, ***P < 0.001).

The enrichment analyses of transcriptome sequencing data showed that DEGs were related to transcriptional factors, membrane-enclosed lumen, as well as pancarcinoma-related pathways and drug resistance (**Figure 3**). Based on the complex mechanism involved in the activation of CAFs and its effect on cancer cells, we proposed that the exosome from CAFs might participate in the modulation by paracrine mechanisms. CAF exosomes contain various components such as proteins, DNA, and RNA that could activate many signaling pathways and facilitate intercellular communication (21). GW4869 is a kind of exosome inhibitor that can decrease CAF exosome secretion by ~70% *in vitro* (24). Also, local injection in the late scar formation period with GW4869 reduced α-SMA^+^ fibroblasts and extracellular matrix production, including collagen I and collagen II (25). The results of exosome inhibition experiments demonstrated that exosome was an important mediator for accelerating the proliferation and migration of A549 cells induced by irradiated CAFs. There was no difference in apoptosis rate (*P* > 0.05) between non-irradiated A549 cells in the G+CAF_0_-CM subgroup and G+CAF_8_-CM subgroup compared with G+DMEM (**Figures 6A, B**). In irradiated A549 cells, the apoptosis rate was lower in the G+CAF_0_-CM subgroup (*P* < 0.001) and G+CAF_8_-CM subgroup (*P* < 0.01) compared with the control.

In the transwell culture system, compared with CAFs, fewer A549 cells could penetrate the polycarbonate membrane regardless of whether the MRC5 cells in the lower chamber underwent ionizing radiation or not, and there was no significant difference in the number of transmembrane cells between the MRC5_0_ subgroup and MRC5_8_ subgroup (*P* > 0.05). The number was about 6 times that of the MRC5_0_ subgroup when under the induction of non-irradiated CAFs (CAF_0_) in the lower chamber (*P* < 0.001). However, ionized CAFs (CAF_8_) induced more A549 cells to migrate downwards, and the number of transmembrane cells was about 1.5 times that of the CAF_0_ group (*P* < 0.001). This result was similar to the migration and the induction of H1299 cells. Nevertheless, when GW4869 was added into the lower chamber, the number of A549 cells induced to transmembrane was significantly lower than that in the non-GW4869 treated subgroup (CAF_0_ or CAF_8_) (*P* < 0.001) regardless if CAFs in the lower chamber were irradiated or not (CAF_0_+G or CAF_8_+G) (**Figures 6C, D**).

### Radiated CAFs promote the deterioration of A549 xenograft

**Figure 7:**
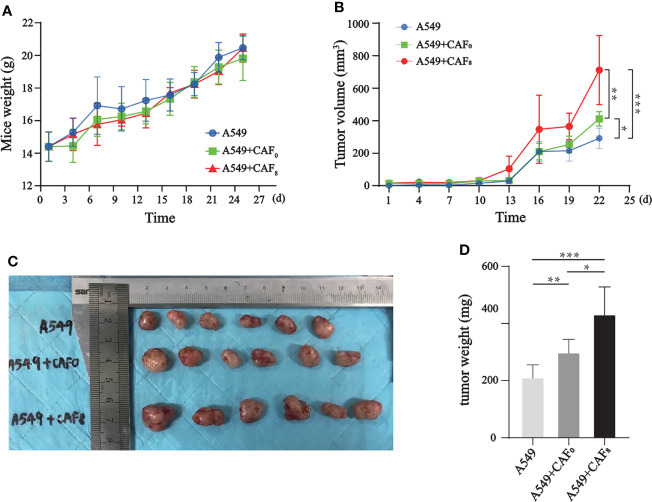
CAFs promoted the tumor volume and weight of the A549 cells tumor model after ionizing irradiation. **(A)** The line graph of mice weight showed that differences of body weight changes for each mouse in different groups was no significant; **(B)** The line graph of tumor volume indicated that CAF_8_ had the greatest promotion on the tumorigenicity of A549 cells; **(C)** An overview of implanted tumors in three subgroups revealed that the tumor volume of the A549+CAF8 group was the biggest, and the A549 group was the smallest; **(D)** The tumor volume was measured for each group of mice and the t-test was applied for statistical analysis. The histogram displayed that CAF8 had the strongest promotion on the tumorigenicity of A549 cells. (T-test, data are presented as the mean ± S.D. *P < 0.05, **P < 0.01, ***P < 0.001).

Mice were subcutaneously inoculated with CAFs (CAF_0_, CAF_8_) and A549 cells with a ratio of 1:1 to establish a subcutaneously implanted tumor model; the control group was inoculated with A549 cells only. All mice successfully developed tumors and gradually gained weight. The rates of weight gain were similar among groups from the slope of the curve (**Figure 7A**). According to the tumor volume growth curve (**Figure 7B**), the volume of implanted tumors in the A549+CAF_0_ group increased faster than in the A549 group (*P* < 0.05), while the A549+CAF_8_ group was the fastest one (*P* < 0.05). After 13 days of inoculation, the volume of implanted tumors in the A549+CAF_8_ group was larger than that in the A549+CAF_0_ and A549 groups (*P* < 0.05). This difference gradually increased in tumor volume among all groups. On the 22^nd^ day of inoculation, the implanted tumor size of several animals reached 15 mm in length (**Figure 7C**). Consequently, all the animals were anesthetized and sacrificed, and *ex vivo* analysis was performed. The tumor of the A549+CAF_8_ group was heavier than the A549+CAF_0_ and A549 group (*P* < 0.05, *P* < 0.001 respectively) (**Figure 7D**). These data indicated that irradiated-CAFs have a stronger promotion effect on the tumor initiation and progression.

**Figure 8:**
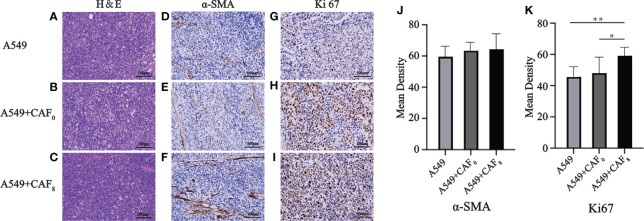
Hematoxylin and eosin (H&E) and immunohistochemical staining of the exfoliated tumor tissues were used to detect histopathological features and expression of α-SMA and Ki67 in A549, A549+CAF_0,_ and A549+CAF_8_ subgroups. **(A–C)** Representative images of H&E staining for three subgroups; **(D–F)** Immunohistochemical staining of α-SMA of three subgroups; **(G–I)**. Immunohistochemical staining of Ki67 of three subgroups; **(J, K)** The histogram of the mean density of α-SMA positive staining area **(J)** and Ki67 positive staining area **(K)** in the samples from all groups. (T-test, data are presented as the mean ± S.D. *P < 0.05, **P < 0.01).

Hematoxylin and eosin (H&E) and immunohistochemical staining suggested that CAFs after ionizing irradiation had higher proliferation ability in A549 transplanted tumors. H&E staining results showed no significant difference in tumor histomorphology among the three groups, which were all distributed in sheet or nest shapes. All tumor cells had obvious atypia, with large and dark nuclei, coarse nuclear chromatin, and mitotic images (**Figures 8A–C**). In comparison to the A549 group, the staining of α -SMA seemed more in the A549+CAF0 and A549+CAF8 groups, but the difference was not significant (**Figures 8D–F, J**). The immunohistochemical results of ki67 also showed that irradiated CAFs promoted the growth of A549 cell tumor and its chromogenic area in the A549+CAF_8_ group was significantly higher than in A549 control (*P* < 0.01) and A549+CAF_0_ group (*P* < 0.05) (**Figures 8G–I, K**).

## Discussion

Radiotherapy (RT) is an important treatment for malignant tumors, and more than 70% of tumor patients receive radiotherapy at different stages of the disease course. However, the emergence of tumor radiotherapy resistance and secondary malignant tumors are matters of utmost importance (26). RT can lead to high load mutations in the tumor genome, and the tumor genome mainly manifests in the form of small fragment deletion, which may be an important mechanism explaining poor prognosis in recent studies (27).

In the RT process, radiation kills tumor cells and affects the TME, forming a special radiation tumor microenvironment (RTME). Moreover, the interaction between tumor cells and TME is also considered a potential factor in inducing RT tolerance (28, 29). Radiation induces a series of interrelated processes in TME, including inflammatory response, local hypoxia, immune regulation, microcirculation reconstruction, interstitial tumor remodeling, and fibrosis (17). Numerous studies have shown that CAFs, a key player in TME, can promote the occurrence, development, metastasis, and formation of treatment tolerance in malignant tumors (10, 11). In the present study, we used A549 and H1299 cell lines as models to validate the effect of irradiated CAFs on tumor cells. Although higher doses of radiation could cause CAFs growth inhibition (30), CAFs irradiated by 8Gy X-rays still had the highest activity (**Figures 1C–E**). Therefore, our subsequent experiments were all based on this radiation dose.

Our results showed that the irradiation of 8Gy X-ray could promote the accelerated replication of nuclei, resulting in double nucleoli, double nuclei, and lobulated nuclei. Moreover, it could increase and expand the rough endoplasmic reticulum and Golgi apparatus in the cytoplasm, indicating that the synthesis ability of proteins, lipids, and sugars was improved. It also increased mitochondrial division, suggesting the enhancement of cellular energy metabolism. The up-regulated expression of α-SMA, FAP, vimentin, and the result of the MTT assay showed that irradiation could enhance the proliferation ability of CAFs. Transcriptome analysis showed a characteristic gene expression profile of radiated CAFs. The oncogenic genes significantly increased after irradiation, and these differentially expressed genes were mainly enriched in several common signaling pathways for promoting cancer progression, including the Ras signaling pathway, PI3K-Akt signaling pathway, and MAPK signaling pathway. CAFs irradiated at a higher dose may show similar genetic changes; however, these cells are prone to growth inhibition due to the activation of p53 signaling and the cell cycle arrest signaling pathway (30).

We further explored the effect of radiation-activated CAFs on lung cancer cells. To simulate the pathophysiological changes of tumors after RT, irradiated A549 cells were used to prepare cancer cell models. Firstly, we found that A549 cells were sensitive to ionizing irradiation, and 6Gy irradiation partially inhibited their growth, while the treatment with a conditioned medium of radiation-activated CAFs could reverse the apoptosis of irradiated A549 cells and promote their growth (**Figure 4C**), thus reflecting the “apoptosis protection” effect of radiation-activated CAFs on cancer cells. These results were further verified in wound healing. Transwell assay demonstrated that radiation-activated CAFs could induce the migration of H1299 cells. Also, these results could help explaining some clinical phenomena, such as local fibrous hyperplasia and distant metastasis induced by RT, where a higher interstitial ratio (TSR) was associated with a worse prognosis. CAFs have a strong radiation tolerance. Although the dose of RT used in clinical practice causes continuous DNA damage, CAFs only undergo senescence and are not eliminated in the tumor. The latter can continue to promote tumor growth and participate in the induction of tumor radiation resistance through the secretion of certain cytokines and growth factors, cooperating with the modified RTME by ionizing radiation (31). The conditioned medium of 8Gy-activated CAFs promoted the migration of A549 cells in the A549-irradiated and non-irradiated groups. The difference among DMEM, CAF_0_-CM and CAF_8_-CM culture groups was greater when A549 received 6-Gy irradiation (**Figure 4B**), and we wonder if there were some special factors that participate in the repair of damaged cancer cells but had no effect on undamaged cells, which could be triggered by DNA breakage or by proteins that related to radiation-induced apoptosis-associated pathway. Nonetheless, the above assumption needs to be further confirmed by subsequent experiments. Moreover, after irradiation of A549, all cells in the irradiated group showed decreased ability to migrate compared to non-irradiated cells, suggesting that activated CAFs could at least partially reverse the damage caused by radiation to cancer cells. However, in the proliferation CCK8 assay, we could also find the difference between DMEM and CAF0-CM in the irradiated group, which indicated that even not activated by irradiation, CAFs exhibited partially pro-malignant behavior, resulting the protection of cancer cells throughout tumorigenesis.

Some previous studies have reported that the release of exosomes is the main way for CAFs to exert the ability to “promote malignancy” (32–34). GW4869 is a cellular permeable, non-competitive N-Smase (neutral sphinomatidylase) inhibitor that inhibits exosome release by blocking neuramide-mediated cellular multivesicular germination (35). In the present study, we obtained similar results by using GW4869. It has also been reported that the exosomes from CAFs can activate the TGF-β signaling pathway in cancer cells and promote the stem-cell nature of colorectal cancer (CRC) cells, thereby increasing radiation resistance and promoting the normal growth of CRC cells in colorectal cancer (36). This was also verified in our transcriptome, which detected upregulation of TGF-β signaling pathways in radiation-activated CAFs (**Figure 3**). Finally, we demonstrated the *in vivo* pro-malignancy effect of radiation-activated CAFs by constructing a rodent model of subcutaneous implantation of tumors. This is similar to previous reports suggesting that CAFs can accelerate the growth rate of implanted cancer cells in animals (13). Our results not only confirmed this conclusion but also found that radiation-activated CAFs have a stronger tumorigenic effect *in vivo* than primary CAFs with regard to tumor size and growth rate.

The previous studies suggested that CAFs have a vital role in the occurrence and development of malignant tumors and the formation of tolerance to RT and chemotherapy; however, few reported on the effects of RT on CAFs (14). Therefore, we proposed that radiation not only kills cancer cells but also transforms CAFs in tumor tissues, named “radiation activation”, thus facilitating cancer cells to survive, migrate, and develop tolerance to RT and chemotherapy. Indeed, TME has undergone great changes compared with ordinary conditions *via* irradiation, which will be beneficial or mediate the qualitative changes of tumor resistance to treatment. Therefore, targeting CAFs or preventing CAFs activation will be beneficial for improving the therapeutic effect of radiotherapy and reducing the occurrence of local tissue fibrosis.

To sum up, the present study confirmed that CAFs could be activated by a certain dose of ionizing radiation and that activated CAFs showed stronger “pro-malignant” biological behavior. Our results also suggested that inhibiting CAFs activation would have a significant effect on improving clinical RT efficacy. With the continuous elucidation of the complicated mechanism as well as the continuous research of CAFs inhibitors or CAFs activation blockers, the CAFs-inhibiting treatment may become a necessary supplement for tumor precision radiotherapy in the future.

## Data availability statement

The original contributions presented in the study are included in the **Supplementary Material**, further inquiries can be directed to the corresponding author (YY).

## Ethics statement

The studies involving human participants were reviewed and approved by Medical Ethics Committee of Renmin Hospital of Wuhan University. The patients/participants provided their written informed consent to participate in this study. The animal study was reviewed and approved by Medical Ethics Committee for Animal Experiments, Renmin Hospital of Wuhan University.

## Author contributions

YY, ZLZ and QS contributed to the conception of the study; ZYZ, YD, BW, YL contributed significantly to carry out experiments and analysis; ZHL and ZML performed the data analyses and wrote the manuscript; YG and LG helped perform the analysis with constructive discussions. All authors contributed to the article and approved the submitted version.

## Funding

This work was supported by Key Youth Training Foundation of Renmin Hospital of Wuhan University (No. 2013-18); Beijing Medical and Health Foundation (No. YWJKJJHKYJJ-BXS5-22006); Chen Xiao-ping Foundation for the Development of Science and Technology of Hubei Province (CXPJJH122006-1005).

## Acknowledgments

We thank Wuhan Frasergen Bioinformatics Co. Ltd for technical support with sequencing and data analysis.

## Conflict of interest

The authors declare that the research was conducted in the absence of any commercial or financial relationships that could be construed as a potential conflict of interest.

## Publisher’s note

All claims expressed in this article are solely those of the authors and do not necessarily represent those of their affiliated organizations, or those of the publisher, the editors and the reviewers. Any product that may be evaluated in this article, or claim that may be made by its manufacturer, is not guaranteed or endorsed by the publisher.

## References

[1] Hoffman D Dragojević I Hoisak J Hoopes D Manger R. Lung stereotactic body radiation therapy (SBRT) dose gradient and PTV volume: a retrospective multi-center analysis. Radiat Oncol (2019) 14:162. doi: 10.1186/s13014-019-1334-9 31481089 PMC6724320

[2] Sato A Rahman NIA Shimizu A Ogita H. Cell-to-cell contact-mediated regulation of tumor behavior in the tumor microenvironment. Cancer Sci (2021) 112(10):4005–12. doi: 10.1111/cas.15114 PMC8486192 34420253

[3] Mao X Xu J Wang W Liang C Hua J Liu J. Crosstalk between cancer-associated fibroblasts and immune cells in the tumor microenvironment: new findings and future perspectives. Mol Cancer (2021) 20:131. doi: 10.1186/s12943-021-01428-1 34635121 PMC8504100

[4] Kalluri R. The biology and function of fibroblasts in cancer. Nat Rev Cancer (2016) 16(9):582–98. doi: 10.1038/nrc.2016.73 27550820

[5] Nomura S. Identification, friend or foe: Vimentin and alpha-smooth muscle actin in cancer-associated fibroblasts. Ann Surg Oncol (2019) 26:4191–92. doi: 10.1245/s10434-019-07894-8 31605319

[6] Borriello L Nakata R Sheard MA Fernandez GE Sposto R Malvar J. Cancer-associated fibroblasts share characteristics and protumorigenic activity with mesenchymal stromal cells. Cancer Res (2017) 77(18):5142–57. doi: 10.1158/0008-5472 PMC5600847 28687621

[7] Fitzgerald AA Weiner LM. The role of fibroblast activation protein in health and malignancy. Cancer Metastasis Rev (2020) 39:783–803. doi: 10.1007/s10555-020-09909-3 32601975 PMC7487063

[8] Primac I Maquoi E Blacher S Heljasvaara R Van Deun J Smeland HY. Stromal integrin alpha11 regulates PDGFR-beta signaling and promotes breast cancer progression. J Clin Invest (2019) 129(11):4609–28. doi: 10.1172/JCI125890 PMC6819106 31287804

[9] Kaps L Schuppan D. Targeting cancer associated fibroblasts in liver fibrosis and liver cancer using nanocarriers. Cells (2020) 9(9):2027. doi: 10.3390/cells9092027 PMC7563527 32899119

[10] Alexander J Cukierman E. Cancer associated fibroblast: Mediators of tumorigenesis. Matrix Biol (2020) 91-92:19–34. doi: 10.1016/j.matbio.2020.05.004 32450219 PMC7434664

[11] Fiori ME Di Franco S Villanova L Bianca P Stassi G De Maria R. Cancer-associated fibroblasts as abettors of tumor progression at the crossroads of EMT and therapy resistance. Mol Cancer (2019) 18:70. doi: 10.1186/s12943-019-0994-2 30927908 PMC6441236

[12] Heichler C Scheibe K Schmied A Geppert CI Schmid B Wirtz S. STAT3 activation through IL-6/IL-11 in cancer-associated fibroblasts promotes colorectal tumour development and correlates with poor prognosis. Gut (2020) 69(7):1269–82. doi: 10.1136/gutjnl-2019-31920 31685519

[13] Su S Chen J Yao H Liu J Yu S Lao L. CD10(+)GPR77(+) cancer-associated fibroblasts promote cancer formation and chemoresistance by sustaining cancer stemness. Cell (2018) 172(4):841–56. doi: 10.1016/j.cell.2018.01.009 29395328

[14] Tommelein J De Vlieghere E Verset L Melsens E Leenders J Descamps B. Radiotherapy-activated cancer-associated fibroblasts promote tumor progression through paracrine IGF1R activation. Cancer Res (2018) 78(3):659–70. doi: 10.1158/0008-5472 29217764

[15] Dumont N Liu B Defilippis RA Chang H Rabban JT Karnezis AN. Breast fibroblasts modulate early dissemination, tumorigenesis, and metastasis through alteration of extracellular matrix characteristics. Neoplasia (2013) 15(3):249–62. doi: 10.1593/neo.121950 PMC3593149 23479504

[16] Sahai E Astsaturov I Cukierman E DeNardo DG Egeblad M Evans RM. A framework for advancing our understanding of cancer-associated fibroblasts. Nat Rev Cancer (2020) 20(3):174–86. doi: 10.1038/s41568-019-0238-1 PMC7046529 31980749

[17] Barker HE Paget JT Khan AA Harrington KJ. The tumour microenvironment after radiotherapy: mechanisms of resistance and recurrence. Nat Rev Cancer (2015) 15:409–25. doi: 10.1038/nrc3958 PMC4896389 26105538

[18] Xia WY Feng W Zhang CC Shen YJ Zhang Q Yu W. Radiotherapy for non-small cell lung cancer in the immunotherapy era: the opportunity and challenge-a narrative review. Transl Lung Cancer Res (2020) 9(5):2120–36. doi: 10.21037/tlcr-20-827 PMC7653139 33209631

[19] Menon H Ramapriyan R Cushman TR Verma V Kim HH Schoenhals JE. Role of radiation therapy in modulation of the tumor stroma and microenvironment. Front Immunol (2019) 10:193. doi: 10.3389/fimmu.2019.00193 30828330 PMC6384252

[20] Verset L Tommelein J Moles Lopez X Decaestecker C Boterberg T De Vlieghere E. Impact of neoadjuvant therapy on cancer-associated fibroblasts in rectal cancer. Radiother Oncol (2015) 116(3):449–54. doi: 10.1016/j.radonc.2015.05.007 26021554

[21] Shi L Zhu W Huang Y Zhuo L Wang S Chen S. Cancer-associated fibroblast-derived exosomal microRNA-20a suppresses the PTEN/PI3K-AKT pathway to promote the progression and chemoresistance of non-small cell lung cancer. Clin Transl Med (2022) 12:e989. doi: 10.1002/ctm2.989 35857905 PMC9299573

[22] Cheng Y Mo F Li Q Han X Shi H Chen S. Targeting CXCR2 inhibits the progression of lung cancer and promotes therapeutic effect of cisplatin. Mol Cancer (2021) 20:62. doi: 10.1186/s12943-021-01355-1 33814009 PMC8019513

[23] Kim CS Kim JM Nam SY Yang KH Jeong M Kim HS. Low-dose of ionizing radiation enhances cell proliferation *via* transient ERK1/2 and p38 activation in normal human lung fibroblasts. J Radiat Res (2007) 48(5):407–15. doi: 10.1269/jrr.07032 17660698

[24] Richards KE Zeleniak AE Fishel ML Wu J Littlepage LE Hill R. Cancer-associated fibroblast exosomes regulate survival and proliferation of pancreatic cancer cells. Oncogene (2017) 36(13):1770–78. doi: 10.1038/onc.2016.353 PMC5366272 27669441

[25] Chen J Zhou R Liang Y Fu X Wang D Wang C. Blockade of lncRNA-ASLNCS5088-enriched exosome generation in M2 macrophages by GW4869 dampens the effect of M2 macrophages on orchestrating fibroblast activation. FASEB J (2019) 33(11):12200–12. doi: 10.1096/fj.201901610 PMC6902732 31373848

[26] Lee SY Jeong EK Ju MK Jeon HM Kim MY Kim CH. Induction of metastasis, cancer stem cell phenotype, and oncogenic metabolism in cancer cells by ionizing radiation. Mol Cancer (2017) 16:10. doi: 10.1186/s12943-016-0577-4 28137309 PMC5282724

[27] Kocakavuk E Anderson KJ Varn FS Johnson KC Amin SB Sulman EP. Radiotherapy is associated with a deletion signature that contributes to poor outcomes in patients with cancer. Nat Genet (2021) 53(7):1088–96. doi: 10.1038/s41588-021-00874-3 PMC8483261 34045764

[28] Ashrafizadeh M Farhood B Eleojo Musa A Taeb S Najafi M. The interactions and communications in tumor resistance to radiotherapy: Therapy perspectives. Int Immunopharmacol (2020) 87:106807. doi: 10.1016/j.intimp.2020.106807 32683299

[29] McLaughlin M Patin EC Pedersen M Wilkins A Dillon MT Melcher AA. Inflammatory microenvironment remodelling by tumour cells after radiotherapy. Nat Rev Cancer (2020) 20(4):203–17. doi: 10.1038/s41568-020-0246-1 32161398

[30] Wang Z Tang Y Tan Y Wei Q Yu W. Cancer-associated fibroblasts in radiotherapy: challenges and new opportunities. Cell Commun Signal (2019) 17:47. doi: 10.1186/s12964-019-0362-2 31101063 PMC6525365

[31] Donlon NE Power R Hayes C Reynolds JV Lysaght J. Radiotherapy, immunotherapy, and the tumour microenvironment: Turning an immunosuppressive milieu into a therapeutic opportunity. Cancer Lett (2021) 502:84–96. doi: 10.1016/j.canlet.2020.12.045 33450360

[32] Luga V Wrana JL. Tumor-stroma interaction: Revealing fibroblast-secreted exosomes as potent regulators of wnt-planar cell polarity signaling in cancer metastasis. Cancer Res (2013) 73(23):6843–7. doi: 10.1158/0008-5472.CAN-13-1791 24265274

[33] Miki Y Yashiro M Okuno T Kitayama K Masuda G Hirakawa K. CD9-positive exosomes from cancer-associated fibroblasts stimulate the migration ability of scirrhous-type gastric cancer cells. Br J Cancer (2018) 118:867–77. doi: 10.1038/bjc.2017.487 PMC5886122 29438363

[34] Xu K Zhang C Du T Gabriel ANA Wang X Li X. Progress of exosomes in the diagnosis and treatment of lung cancer. BioMed Pharmacother (2021) 134:111111. doi: 10.1016/j.biopha.2020.111111 33352449

[35] Dinkins MB Dasgupta S Wang G Zhu G Bieberich E. Exosome reduction *in vivo* is associated with lower amyloid plaque load in the 5XFAD mouse model of alzheimer's disease. Neurobiol Aging (2014) 35(8):1792–800. doi: 10.1016/j.neurobiolaging.2014.02.012 PMC4035236 24650793

[36] Liu L Zhang Z Zhou L Hu L Yin C Qing D. Cancer associated fibroblasts-derived exosomes contribute to radioresistance through promoting colorectal cancer stem cells phenotype. Exp Cell Res (2020) 391(2):111956. doi: 10.1016/j.yexcr.2020.111956 32169425

